# Bacterial Competition Influences the Ability of Symbiotic Bacteria to Colonize Western Flower Thrips

**DOI:** 10.3389/fmicb.2022.883891

**Published:** 2022-07-07

**Authors:** Awawing A. Andongma, Miranda M. A. Whitten, Ricardo Del Sol, Matthew Hitchings, Paul J. Dyson

**Affiliations:** Applied Molecular Microbiology Group, Institute of Life Sciences, Swansea University School of Medicine, Swansea, United Kingdom

**Keywords:** symbiotic bacteria, Western Flower Thrips, bacterial competition, paratransgenesis, next-generation sequencing

## Abstract

Symbiont mediated RNAi (SMR) is a promising method for precision control of pest insect species such as Western Flower Thrips (WFT). Two species of bacteria are known to be dominant symbiotic bacteria in WFT, namely BFo1 and BFo2 (Bacteria from *Frankliniella occidentalis* 1 and 2), as we here confirm by analysis of next-generation sequence data derived to obtain a reference WFT genome sequence. Our first demonstration of SMR in WFT used BFo2, related to *Pantoea*, isolated from a domesticated Dutch thrips population. However, for successful use of SMR as a thrips control measure, these bacteria need to successfully colonize different environmental thrips populations. Here, we describe a United Kingdom thrips population that does not harbour BFo2, but does contain BFo1, a species related to *Erwinia*. Attempts to introduce BFo2 indicate that this bacterium is unable to establish itself in the United Kingdom thrips, in contrast to successful colonization by a strain of BFo1 expressing green fluorescent protein. Fluorescence microscopy indicates that BFo1 occupies similar regions of the thrips posterior midgut and hindgut as BFo2. Bacterial competition assays revealed that a barrier to BFo2 establishing itself in thrips is the identity of the resident BFo1; BFo1 isolated from the United Kingdom thrips suppresses growth of BFo2 to a greater extent than BFo1 from the Dutch thrips that is permissive for BFo2 colonization. The ability of the latter strain of BFo1 to colonize the United Kingdom thrips is also likely attributable to its ability to out-compete the resident BFo1. Lastly, we observed that United Kingdom thrips pre-exposed to the Dutch BFo1 could then be successfully colonized by BFo2. These results indicate, for the first time, that microbial competition and strain differences can have a large influence on how symbiotic bacteria can colonize different populations of an insect species.

## Introduction

The number of known insect species exceeds 1 million, and only for a very small proportion of these do we have some idea about specific associations with symbiotic bacteria (Ferrari and Vavre, 2011). There is a spectrum of known symbiotic relationships. These range from examples of obligate endosymbionts that, for example, provide their insect hosts specific nutrients, exemplified in the case of aphids harboring *Buchnera* species (Akman Gunduz and Douglas, 2009), to facultative symbionts that are beneficial but not essential to their host, and whose abundance may reflect particular environmental conditions the host encounters. An example of the latter is the protection afforded to aphids by *Hamiltonella defensa* against parasitoid wasps that comes at a cost to insect fitness in the absence of parasitism (Vorburger et al., 2013).

The spectrum of symbioses is also reflected in that, for specific host–microbe interactions, different tissues can be colonized by bacteria and the mechanisms for how symbiotic bacteria are transmitted are varied. Some insect species possess specific cells such as bacteriocytes to “house” their bacteria and these microbes are often described as obligate endosymbionts (Baumann, 2005), whereas for other insect species their symbiotic bacteria can simply be dominant members of the gut microbiome, as in the case of hemipteran triatomines (Salcedo-Porras et al., 2020). For the latter, mechanisms such as coprophagy ensure transgenerational transmission (Beard et al., 2001), whereas obligate symbionts are frequently known to be vertically transmitted, as exemplified in aphids (Koga et al., 2012).

An example of an insect where there is a well-documented example of specific bacterial association is the Western Flower Thrips (WFT), *Frankliniella occidentalis*. WFT is a globally-invasive agricultural pest of significant importance (Reitz et al., 2020). The insects cause direct damage to crops by feeding and/or ovipositing on their leaves and fruits which eventually results in a decreased yield and low market value. They also transmit plant pathogenic Tospoviruses (including Tomato Spotted Wilt Virus) which causes significant economic damage to plants (He et al., 2020). WFT is a host to two facultative symbionts named Bacteria of *F. occidentalis* 1 and 2, (BFo1 and BFo2; de Vries et al., 2001a; Chanbusarakum and Ullman, 2008; Facey et al., 2015). The relationship between the thrips and bacteria was first reported in 1989 (Ullman et al., 1989), although these symbionts were identified from thrips collected and preserved in ethanol since 1965 (Chanbusarakum and Ullman, 2008). In addition, BFo1 & 2 have been isolated from different geographic locations including California, Hawaii, Germany, Netherlands, and the United Kingdom (de Vries et al., 2001a; Chanbusarakum and Ullman, 2008, 2009; Facey et al., 2015). This evidence implies a symbiotic rather than a transient relationship. Previous analysis of the distribution of these bacteria in different thrips populations indicated that colonization by BFo1 may be influenced by environmental condition such as temperature, humidity and altitude (Chanbusarakum and Ullman, 2009). This bacterium is related to *Erwinia* spp. (Facey et al., 2015). Colonization by BFo2, on the other hand, has been suggested to be influenced by the sex of the insects, with more males having BFo2 than females (Chanbusarakum and Ullman, 2009). BFo2 is a novel species related to *Pantoea* (Facey et al., 2015). Transmission studies indicate that thrips larvae acquire these bacteria *via* feeding, likely through plant material contaminated with the frass of adults (de Vries et al., 2001b).

RNA interference targeting essential genes is increasingly being applied for insect pest control (Christiaens et al., 2020), but is constrained by how interfering RNA can be delivered to many species. Strategies that can be scaled up for field applications depend on insects acquiring interfering RNA in their diets from, for example, transgenic crop plants expressing double-stranded (ds) RNA (Head et al., 2017). However, due to the activity of RNases in the upper digestive tract, many insect species can evade the lethal effects of ingested dsRNA, as is the case for Western Flower Thrips (Andongma et al., 2020). The use of symbiotic bacteria to deliver dsRNA represents a possible alternative (Whitten et al., 2016; Whitten and Dyson, 2017), whereby the dsRNA is synthesized *in situ* by bacteria that colonize the gastrointestinal (GI) tract. This approach is termed symbiont-mediated RNAi (SMR).

Previously, we demonstrated that BFo2 can be used for delivery of lethal dsRNA to domesticated Dutch thrips on a population scale without manipulating individual insects (Whitten et al., 2016; Whitten and Dyson, 2017). However, optimization of this approach as a general control strategy requires a better understanding of factors that influence the ability of BFo2 to colonize thrips. In this study, we have investigated the symbiotic bacteria of a thrips population collected from a United Kingdom strawberry field. We show that BFo2 could not be detected in these insects and even after experimental introduction, the bacterium is unable to establish itself in these insects. In contrast, BFo1 is present in these thrips and we demonstrate that it colonizes a similar niche in the gut to that which can be occupied by BFo2. Moreover, our data indicate that BFo1 strain differences have an direct impact on the success of colonization by BFo2.

## Materials and Methods

### Insects

The insects used for this study were collected from a strawberry farm in Hereford, United Kingdom (grid ref.: 51.952409,-2.652005) during the summer of 2018. They had been maintained under controlled conditions (23°C ± 2°C, 60–80% relative humidity and a light: dark cycle of 14 h:10 h) at Swansea University on non-organic chrysanthemum plants and runner beans.

### Isolation and Identification of Symbiotic Bacteria

Prior to surface-sterilization, thrips were immobilized by placing them on Parafilm spread over a cold block under a stereomicroscope. Insects were surface-sterilized by immersion in a drop of iodine for 3 to 5 min followed by 3 rinses in sterile MilliQ water. Homogenates of sterilized insects were plated on Luria-Bertani (LB) medium and incubated at 30°C for 48 h. The final insect rinse was also plated to ensure that surface-sterilization was thorough. DNA was isolated from thrips using a Quick-DNA^™^ Tissue/Insect Microprep kit (Zymo Research) following the manufacturer’s recommended protocol. BFo1 and 2 species identification was confirmed by PCR using the BFo-specific primers listed in Table 1 under the following conditions: 95°C for 5 min, followed by 34 cycles of denaturation at 95°C for 30 s, annealing at 52.5°C for 30 s and extension at 72°C for 1 min, and a final extension at 72°C for 5 min.

**Table 1:** Bacterial strains and plasmids used in this study.

Strain or plasmid	Relevant properties	Reference or source
BFo1-D	BFo1 isolated from Dutch WFT population	Facey et al., 2015
BFo2-D	BFo2 isolated from Dutch WFT population	Facey et al., 2015
BFo1-H	BFo1 isolated from a UK thrips population	This study
*E. coli* JM109	Standard cloning host	Yanisch-Perron et al., 1985
pdag-GFP	Multicopy copy ampicillin resistant GFP-expressing plasmid	This study
pMK	Multicopy copy kanamycin resistant plasmid	GeneArt (ThermoFisher)

### Plasmids

Plasmids and bacterial strains are listed in Table 1. The plasmid pdag-1 (Whitten et al., 2016; Dyson et al., 2022), conferring resistance to ampicillin, was used to construct a derivative encoding GFP. The GFP gene was obtained by PCR amplification using primers GFP-RVF and GFP-RVR (Table 2) and plasmid pUC18T-mini-Tn7-Zeo-gfpmut3 (Choi and Schweizer, 2006) as template. Both the amplicon and pdag-1were cut with EcoRV, and the latter then dephosphorylated with Quick CIP (NEB), before ligation, to derive pdag-GFP. To construct a kanamycin-resistant version of BFo1-D, the multicopy copy plasmid pMK (GeneArt) was introduced. Plasmids were introduced into both BFo1 and BFo2 by electroporation (Dyson et al., 2022). Plasmid stability was assessed by passaging the bacteria in non-selective LB broth. At different time intervals over a two week period, serial dilutions of cultures were plated in parallel on non-selective and selective LB agar plates (ampicillin 100 μg/ml; kanamycin 50 μg/ml), prior to colony counting the following day.

**Table 2:** PCR primers.

Purpose	Name	Sequence (5′…)	Size of amplicon
Detection of BFo1	BFo1PEG1885sF	GATCAACCCGCGCTGTTATC	200 bp
	BFo1PEG1885sR	CTGGCGGATATCCTCAACCA	
Detection of BFo2	BFo1PEG1526MF	CTGGAGCCCTGAAGTGAGAA	350 bp
	BFo1PEG1526MR	CTGCTCACTCTCTGGGTTGA	
Amplification of GFP	GFP-RVF	AGTACTGATATCCACTAGTGAGCTCGTTGCGC	805 bp
	GFP-RVR	GTCAATGATATCCAACAGGAGTCCAAGCTCAGC	

### Bfo1 and 2 Colonization of the Western Flower Thrips

To investigate BFo1 and 2 colonization patterns, bacteria were introduced *via* an artificial feeding solution as previously reported (Andongma et al., 2020). BFo1 or BFo2 strains possessing pdag-GFP were added to the diet as previously described (Whitten et al., 2016). Twenty insects (10 first- or second-instar larva and 10 adults) were placed into each of three glass vials; after their collection from the Hereford strawberry farm, these insects were maintained on chrysanthemum plants, as described for our previous work with a Dutch WFT population. No steps were taken to eliminate or reduce the resident bacteria prior to exposure to the artificial feeding solution. On days 2, 4 and 6 after exposure to the artificial feeding solution, a vial was opened, and all 20 insects were individually surface-sterilized, homogenized, and serial dilutions plated on ampicillin-containing LB plates. Only living insects were sampled, and if any insects died during the experiment (typically losses were < 10%), the numbers were made up by replacing the dead individuals by insects at the same developmental stage from ‘reserve’ vials of the relevant treatment group. Plates were incubated for 24–48 h at 30°C. Successful colonization was recorded if >150 colony forming units (CFUs) of the specific bacterial species were recovered from an undiluted homogenate from an individual insect. Typically, > 20 times this number of CFUs were recovered from successfully colonized insects The identity of the bacterial colonies was confirmed using wide-field fluorescence microscopy (Leica) to monitor expression of green-fluorescent protein.

### Microscopy

Thrips were anaesthetized and examined using fluorescence microscopy as previously described (Whitten et al., 2016).

### Bacterial Competition Assay

We adapted an assay designed to monitor Type VI secretion system-mediated bacterial competition (Hachani et al., 2013). Equal volumes of stationary phase cultures with similar OD_600_ values (equivalent to 10^8^ cells/ml) of the predator bacterial strain (plasmid-free) and prey (containing pdag-GFP) were mixed in a microfuge tube, and 20 μl plated as a single spot on the surface of an LB agar plate. In parallel, 10 μl of the individual predator and prey strains were spotted on LB agar plates. After drying the spots, plates were incubated for 5 h at 30°C. The bacteria from each spot were collected, resuspended in phosphate-buffered saline, and serial dilutions prepared that were then plated in parallel on LB agar plates containing ampicillin and LB plates without antibiotic. The numbers of colonies were enumerated after 24 h incubation at 30°C.

### Taxonomic Assessment of *Frankliniella occidentalis* Microbiota From Existing Sequence Data

A custom Kraken2 database (Wood et al., 2019) was constructed to encompass the following NCBI RefSeq categories: bacteria, archaea, fungi, plant, protozoa, viral and plasmid using the Kraken2-build process. Additionally, the genomes of BFo1 (GenBank Accession: GCA_001602545.), BFo2 (GenBank Accession: GCA_001602605.1) and *F. occidentalis* (GenBank Accession: GCA_000697945.4) were also added to the library with existing NCBI taxonomy IDs used: NCBI:txid1628855, NCBI:txid1628856 and NCBI:txid133901, respectively. Short paired-end sequence reads from the reference *F. occidentalis* hologenome sequence, BioProject accession PRJNA203209, (285,047,552 in total) were queried against the custom Kraken2 database. Graphical representation of read proportions were generated using the R packages cowplot, ggforce and tidyverse.

### Statistical Analysis

Statistical analyses were carried out using Graphpad Prism version 8 or in Excel. Colonization data was analyzed using Fisher’s exact test, the categorical variable being colonization by either BFo1 or BFo2. Statistical analysis of bacterial competition data used unpaired t-tests (2-tailed) after checking homogeneity of variances using *F* tests. Values for these variances were:

o1D/o2 vs. o1H/o2: *F* test gives *p* = 0.1007.
o1D/o1H vs. o1H/o1D: *F* test gives *p* = 0.3111.
o1D/Ec vs. o1H/Ec: *F* test gives *p* = 0.4114.

## Results

### The Prevalence of BFo1 in WFT

**Figure 1:**
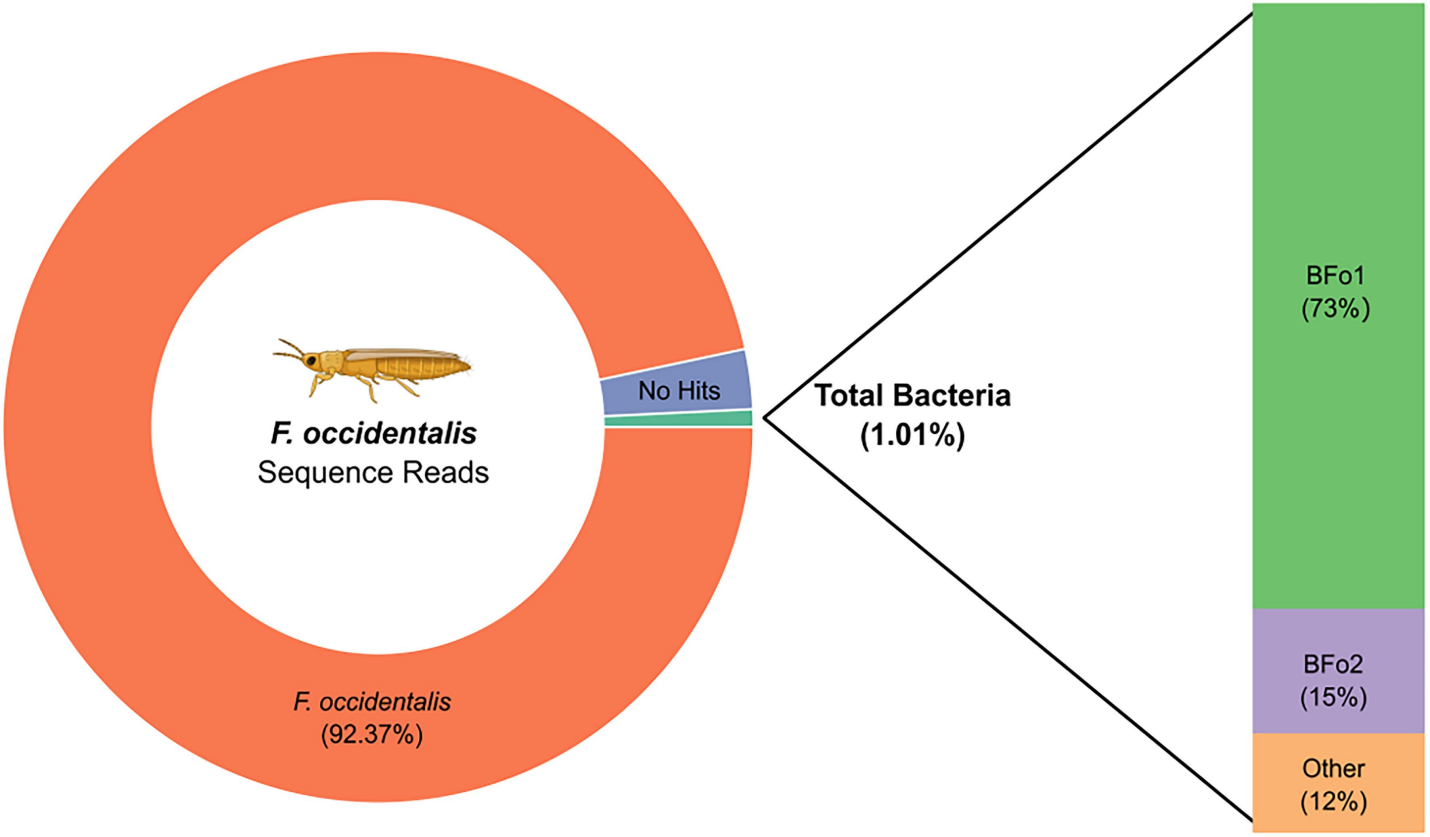
Graphic representation of the distribution of sequence reads from the reference WFT genome sequencing project.

One way to assess the abundance of a specific bacterial species in an insect population is to interrogate next-generation sequence data derived from that insect population prior to removal of non-insect DNA sequences. Interrogation of the 285 million publicly available sequence reads derived from pools of adult WFT that were used to obtain a reference genome sequence revealed the presence of reads of bacterial origin. As expected, 92.37% of total sequence reads (263,286,903) were assigned explicitly to *F. occidentalis*. The number of unclassified reads totaled 2.46% (7,009,103 reads). However, a total of 1.01% of reads (2,887,868) were assigned to the Kingdom Bacteria (NCBI:txid2). Of these bacterial reads, a substantial proportion, 0.73% of total reads (2,069,193 reads) were assigned to the symbiont BFo1 (NCBI:txid1628855; Figure 1). This equates to 71.65% of all bacterial reads derived from a single bacterial species. About 15% of reads were derived from BFo2. Consequently, in the thrips population used to obtain the reference genome sequence, BFo1 and BFo2 were by far the most abundant bacteria, with the former approximately five times more abundant than the latter. One caveat to this approach is that it does not inform on the abundance of these bacteria in individual adult insects, but reflects the pool of insects used for DNA extraction. In addition, it does not describe the bacteria associated with non-adult forms.

**Figure 2:**
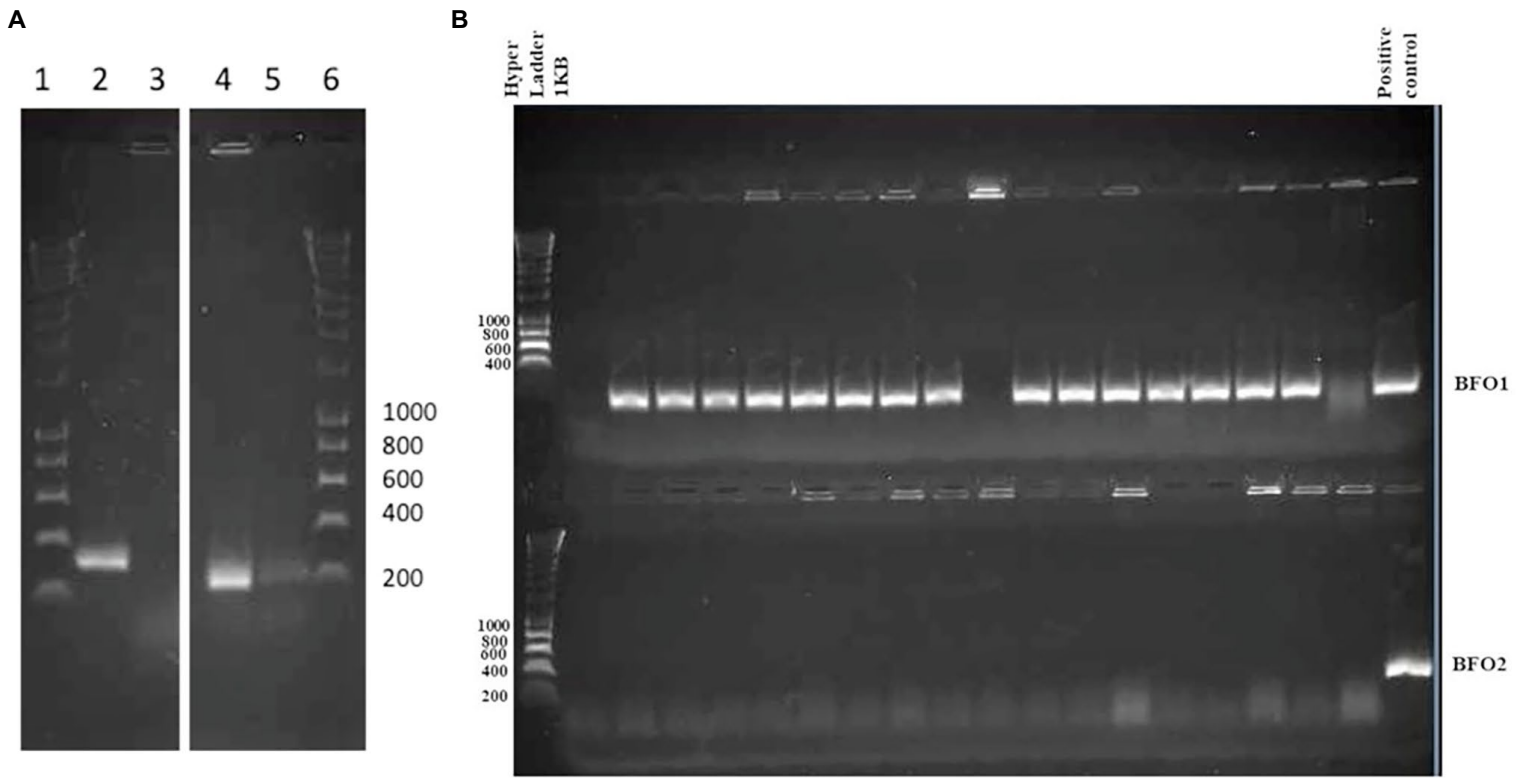
**(A)** Detection of only BFo1 in Hereford-WFT. BFo2-specific primers were used in PCRs with BFo2 DNA (lane 2) and Hereford-WFT DNA (lane 3); BFo1-specific primers were used in PCRs with BFo1 DNA (lane 4) and Hereford-WFT DNA (lane 5). Sizes (bp) of DNA fragments in a 1 kb Hyperladder (lanes 1 and 6) are indicated. **(B)** Representative individual colony PCR analysis of culturable bacteria obtained from Hereford-WFT. PCR reactions with BFo1-specific primers were run on the top half of the gel together with a positive control, as indicated. PCR reactions with BFo2-specific primers were run on the bottom half of the gel together with a positive control, as indicated. Sizes (bp) of DNA fragments in a 1 kb Hyperladder (lanes 1 in top and bottom) are indicated.

Of interest is whether this distribution of bacteria is representative of not only domesticated and possibly inbred thrips colonies, but also environmental populations. Consequently, we collected thrips from a United Kingdom strawberry farm (Hereford-WFT). DNA was extracted from a pool of 40 adult insects, and PCR used to attempt to detect both BFo1 and BFo2. While a diagnostic amplicon for BFo1 was observed, no BFo2- specific amplicon was detected (Figure 2A). Moreover, after plating insect homogenates derived from 20 surface-sterilized adult insects on agar plates and analyzing 150 randomly chosen colonies, 88% of the culturable colonies that grew were BFo1, as confirmed by colony PCR (Figure 2B), whereas no BFo2 bacteria could be detected in these homogenates of the Hereford-WFT population. The typical CFU count per insect homogenate was between 2 × 10^3^ and 5 × 10^3^.

### Stable Colonization of Hereford-WFT by BFo1 but Not BFo2

We previously reported successful colonization of a long-term domesticated Dutch-WFT non-axenic population with BFo2 (Whitten et al., 2016). We had initially isolated the original strain of this BFo2, hereafter termed BFo2-D, from the same Dutch-WFT (Facey et al., 2015). In the current study, we introduced a plasmid (pdag-GFP) encoding the GFP gene into a strain of BFo1 isolated from the same Dutch-WFT population (hereafter termed BFo1-D) and also BFo2-D. The stability of the plasmid encoding GFP was assessed by passaging the recombinant strains of BFo1-D and BFo2-D *in vitro* in growth medium with no selection for retention of the plasmid. No loss of plasmid was detected after approximately 100 generations of either bacterial species, equivalent to 14 days of passaging. We then compared the ability of both these species to colonize Hereford-WFT *via* either continual exposure to an artificial feeding solution in which the bacteria were introduced or limited exposure for just 2 days to the feeding solution containing the bacteria.

**Figure 3:**
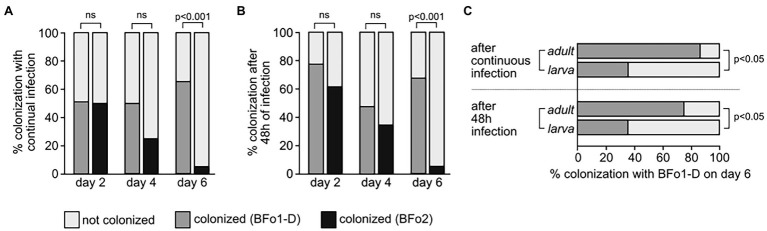
Colonization of Hereford-WFT by BFo bacteria. Thrips were challenged with an artificial feed combined with either BFo1-D or BFo2 continuously **(A)**, or for 2 days **(B)**. In a separate experiment, the proportion of adults and larvae successfully colonized by BFo1-D was determined **(C)**. By Day 6, larvae had metamorphosed into pupae, but none had emerged as adults. ns: no significant difference.

These experiments demonstrated that by either continual or limited exposure, BFo1-D colonized approximately 50% of the thrips population overall, and proliferated in the thrips gut for more than 6 days post feeding. In contrast, although BFo2-D was detected in a similar percentage of thrips at 2 days post-feeding, the the proportion of colonized insects rapidly declined, and by day 6 the majority (~ 95%) of the insects had lost BFo2-D (Figures 3A,B). As such, significantly more insects were colonized by BFo1-D compared to BFo2-D by day 6 (*p* < 0.001 for both continuous feeding and 2-day feeding). When life stages were compared, the results indicated that BFo1-D proliferated in more adults than in larvae by day 6 (Figure 3C; *p* = 0.0135 for continuous feeding, and *p* = 0.0351 for 2-day feeding).

Of the insects successfully colonized by BFo1-D, the great majority (on average 82%) yielded several thousand GFP-expressing CFUs per undiluted body homogenate per plate, i.e., well above the minimum threshold of 150 CFUs. This high number of recovered BFo1-D CFUs was equivalent to CFU numbers of BFo1-H bacteria from the homogenates of untreated insects. Moreover, the number of CFUs from these homogenates growing on plates without added antibiotic were similar to the number growing on antibiotic-containing plates, suggesting BFo1-D could potentially displace the resident BFo1-H in a large proportion of treated insects.

### BFo1 Colonizes the Same Niche in the GI Tract as BFo2

**Figure 4:**
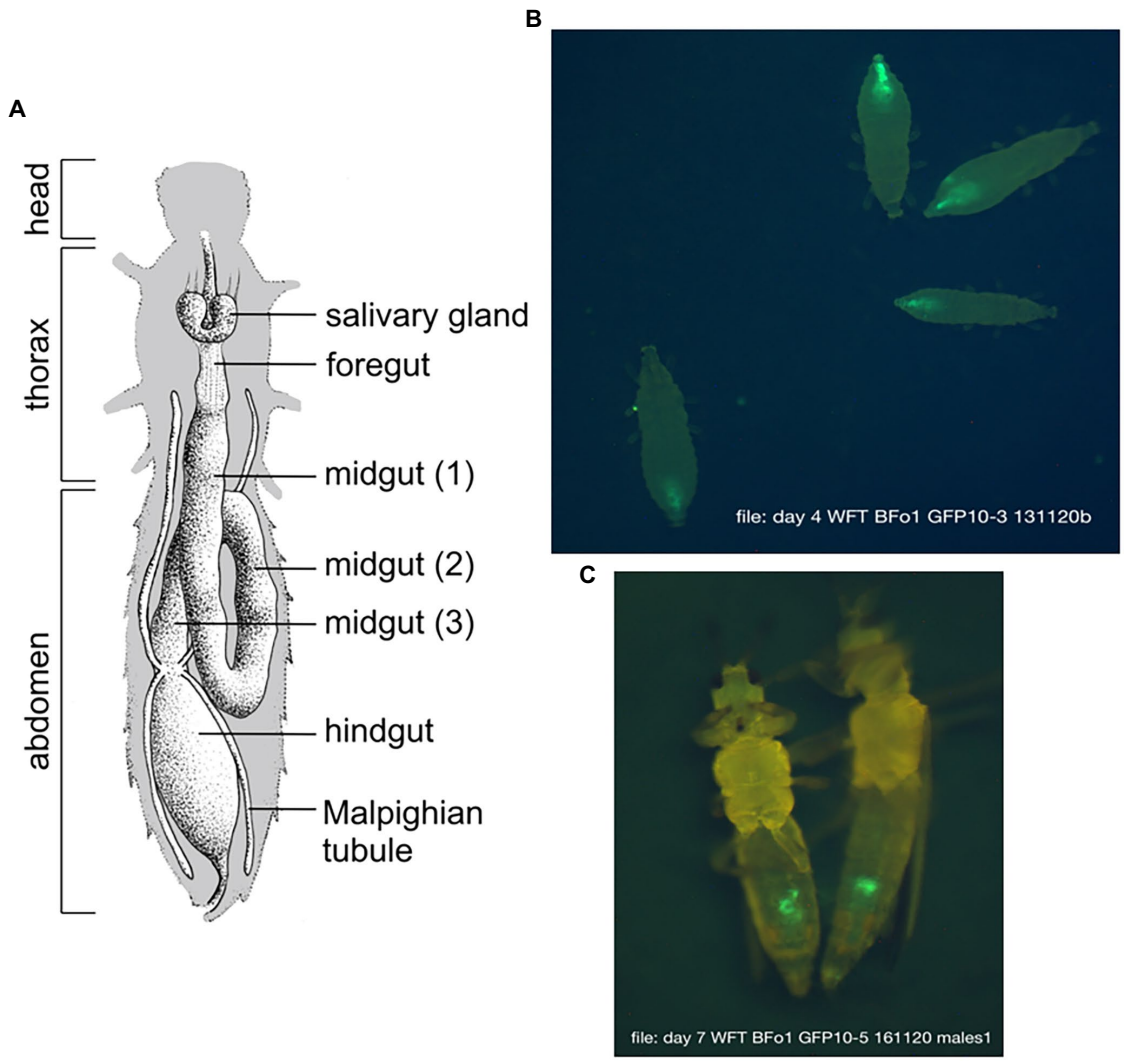
Localisation of BFo1 in WFT larvae and adults. **(A)** Graphical depiction of the anatomy of the GI tract of WFT. **(B)** Fluorescence microscopy of 4 representative WFT larvae indicating colonization of the respective hindguts by BFo1-D expressing GFP, 4 days after feeding. **(C)** Two representative adult males, indicating colonization of the posterior midgut by BFo1-D expressing GFP, 7 days after feeding.

We previously established that GFP-expressing BFo2-D colonizes the posterior midgut and hindgut of Dutch-WFT (Whitten et al., 2016; Whitten and Dyson, 2017). Here, we again employed fluorescence microscopy to examine which region of the gut of the Hereford-WFT was populated by GFP-expressing BFo1-D. In both larvae and adults, we detected the bacteria in the hindguts of larvae and posterior midguts of adult males (Figures 4A–C). The localization of the bacteria in adult females was less easy to define precisely due to autofluorescence emitted by oocytes in the posterior abdomen.

### BFo2-D Is Susceptible to BFo1-H in an *in vitro* Competition Assay

**Figure 5:**
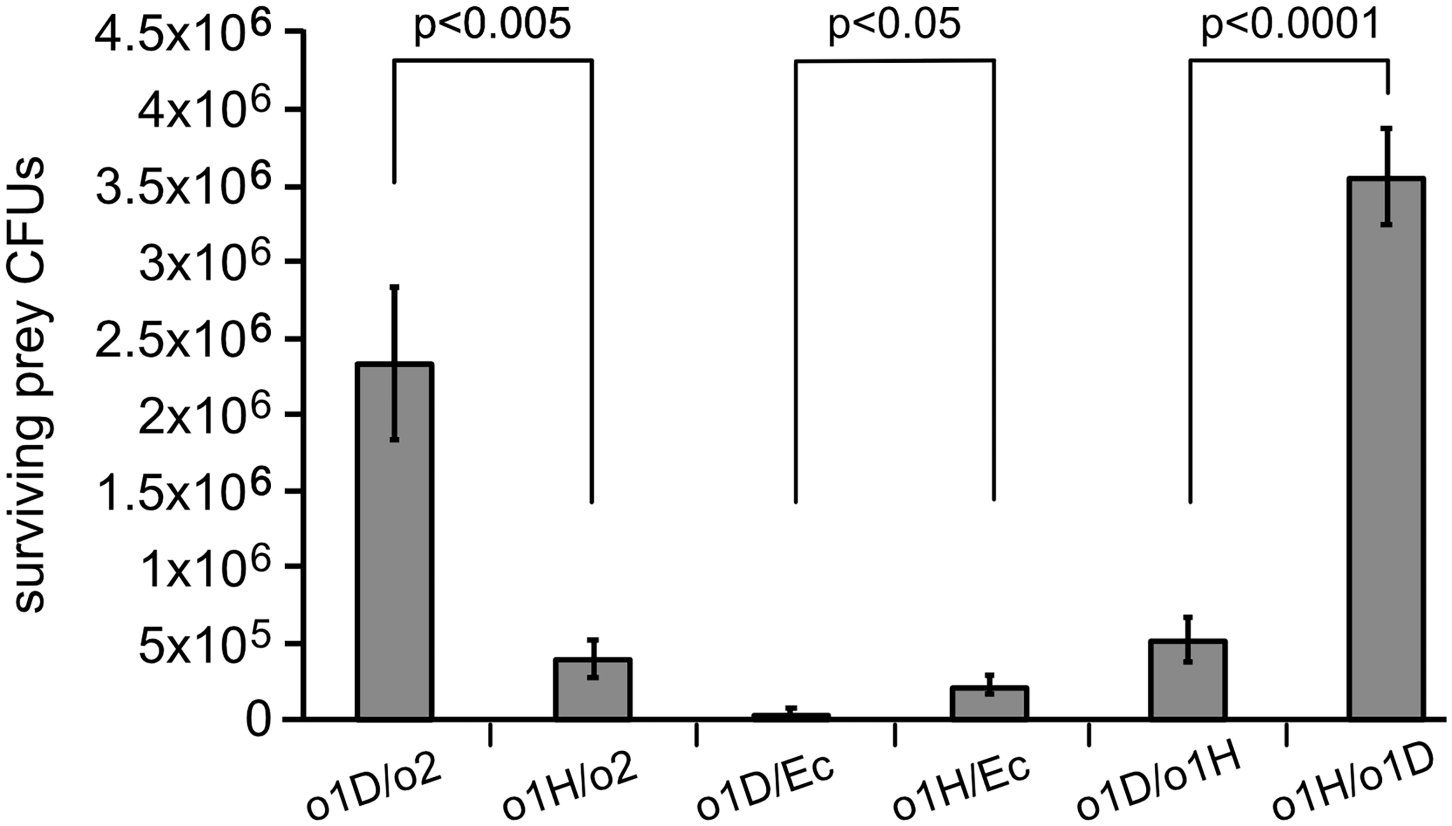
Quantification of the killing of prey bacteria after pairwise competition. The graph represents the surviving CFU counts of prey bacteria after pairwise competition assays. Each pair is indicated as predator/prey for BFo1-D (o1D), BFo1-H (o1H), BFo2 (o2) and *E. coli* (Ec). The data are derived from three replicate experiments for each pair. The average CFUs recovered under non-challenged conditions for each strain were: BFo1-D 1.5 × 10^7^; BFo1-H 1.4 × 10^7^; BFo2 1.8 × 10^7^ and *E. coli* 1.9 × 10^7^.

The lower initial efficiency of colonization of the Hereford-WFT by BFo2-D and its subsequent loss could be related to genetic differences between this recently established thrips colony and the longer-established and possibly inbred Dutch-WFT population. Alternatively, it could also be in part due to differences between the resident BFo1 bacteria present in the two different thrips populations. To address the latter, we isolated BFo1 from the Hereford-WFT, hereafter termed BFo1-H. Bacterial competition assays have previously indicated that commensal bacteria have the ability to suppress the growth of other microbes that compete to occupy similar niches (Kwong and Moran, 2015; Ortiz et al., 2021). Consequently, we carried out *in vitro* bacterial competition assays between the different strains and species of bacteria from the Dutch and Hereford WFT populations (Figure 5). We also compared interbacterial competition with the two different BFo1 strains as ‘predators’ and *E. coli* as a surrogate ‘prey’. The results indicated that during the 5 h period of contact, BFo1-H was able to kill BFo2-D to a 5-fold greater extent than does BFo1-D (*p* < 0.005; Figure 5). In contrast, while incubation of either BFo1 strain with *E. coli* as prey resulted in ≥90% killing, BFo1-D was measurably more successful at killing than BFo1-H (Figure 5, *p* < 0.05). To analyse if successful colonization of Hereford-WFT by BFo1-D could also be attributed to its ability to out-compete the resident BFo1-H, we then conducted parallel competition assays in which each strain was the prey. This revealed that BFo1-D was significantly superior at killing BFo1-H (*p* < 0.0001) than vice versa.

### Pre-colonization of Hereford-WFT With BFo1-D Permits Subsequent Colonization by BFo2-D

**Figure 6:**
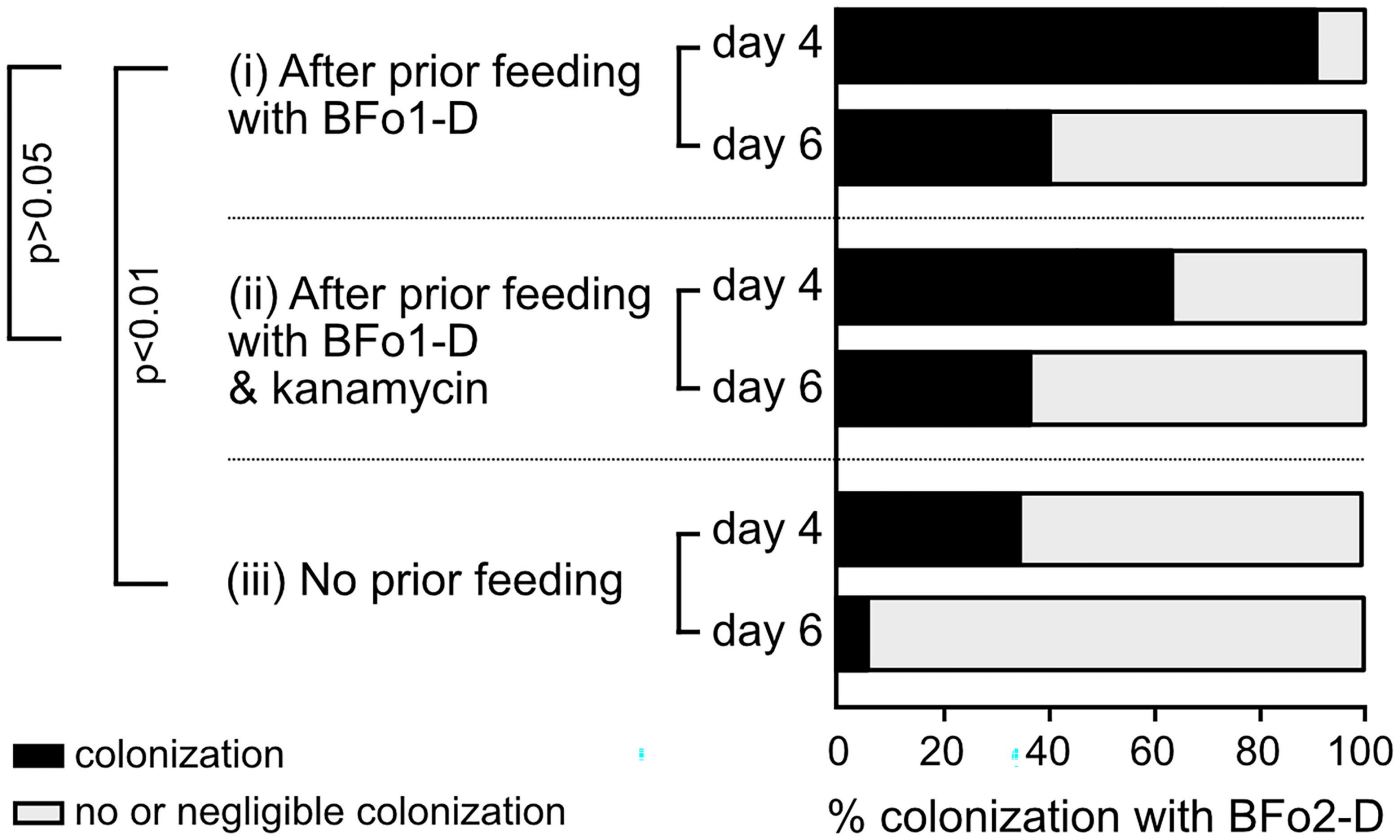
The impact of pre-exposure of adult Hereford-WFT with BFo1-D, on subsequent colonization by BFo2-D. The percentage of Hereford-WFT colonized by BFo2-D was determined 4 and 6 days after oral inoculation with BFo2-D, which followed after an initial 2-day exposure to a feeding solution containing (i) BFo1-D alone, or (ii) BFo1-D with kanamycin. The colonization of Hereford-WFT by BFo2-D, in the absence of pre-exposure, is also shown for comparison (iii).

We rationalized that by displacing BFo1-H with BFo1-D in Hereford-WFT we could determine the relative contributions of the host and the different BFo1 strains to preventing colonization by BFo2-D. Consequently, we first introduced a strain of BFo1-D resistant to kanamycin into Hereford-WFT by feeding for 2 days, following the colonization protocol described above except that only adult insects were treated. We tested two different strategies: exposure of the thrips to a feeding solution containing (1) BFo1-D alone, and (2) BFo1-D and kanamycin. In both cases, we could successfully recover a similar proportion of insects from which kanamycin-resistant BFo1-D could be isolated as an abundant resident bacterium as we had previously observed after colonization by BFo1-D expressing GFP. We then assessed the efficiency for BFo2-D expressing GFP to colonize these thrips now containing BFo1-D, again using the colonization protocol described. The results indicated that BFo2-D could successfully establish itself in Hereford-WFT that had previously been exposed to BFo1-D without kanamycin addition, with 41% of adult insects possessing BFo2-D 6 days after feeding, compared to 6% of adults that had not been pre-exposed to BFo1-D. Inclusion of kanamycin had no major difference on the subsequent ability of BFo2-D to colonize insects pre-exposed to BFo1-D, with 36% of adults possessing BFo2-D in this treatment group 6 days after feeding (Figure 6). These data indicate that the specific strain of BFo1 occupying the WFT gut has a major influence on the ability of BFo2-D to colonize the same niche in these insects.

## Discussion

Although relatively few specific insect-microbe symbioses have been studied in any depth to date, these investigations have revealed that the nature of these symbioses is very diverse and the benefits for the host quite varied (Engel and Moran, 2013; Douglas, 2015; Frago et al., 2020). It is also possible to exploit endosymbiotic bacteria for human benefit, a prime example being the use of intracellular *Wolbachia* bacteria in mosquito populations to suppress transmission of arboviruses such as the Dengue virus (Gabrieli et al., 2021). In addition, strategies such as paratransgenesis, using genetically modified symbiotic bacteria, can be used to modify the biology of the host insect (Elston et al., 2021). An example of the latter is SMR, whereby expression of a target host gene can be reduced due to production of interfering RNA by the symbiotic bacteria such as BFo2 in WFT (Whitten and Dyson, 2017).

Our analysis of the reference WFT genome sequence data, prior to removal of the non-insect DNA sequences, confirms that this insect possesses two dominant bacterial symbionts, BFo1 and BFo2. Indeed, it is worth noting that this type of analysis is applicable to any sequenced animal genome and can reveal the identity of both culturable and non-culturable bacterial symbionts. To date, a study of the impact of these two symbionts of WFT on the biology of their host has been limited to the diet of the insect. Comparing WFT with and without their symbiotic bacteria, the aposymbiotic insects were observed to develop faster and be more fecund on a mixed diet of cucumber leaves and pollen, whereas they were slower in their development and produced fewer eggs on a diet of only cucumber leaves (de Vries et al., 2004).

Our analysis of the Hereford WFT population, however, indicates that not only is BFo2 absent but also it cannot stably colonize these insects after experimental introduction. While we cannot exclude that geographically separated insect populations have genetically diverged and that this could contribute to small differences in supporting BFo2 colonization, our analyses indicate that strain differences between BFo1 symbionts from different insect populations play a major role in this process. Critically, by displacing the resident BFo1-H strain in the Hereford WFT population with BFo1-D, a strain known to co-exist with BFo2 in a Dutch WFT population, the Hereford WFT then became permissive for BFo2 colonization. This suggests that BFo1 strain differences, rather than differences between host populations, are more critical in terms of BFo2 colonization.

The two bacterial species occupy the same niche in the insects: the hindgut of larvae and the posterior midgut of adults. Sharing this niche provides an environment where competitive mechanisms can shape the bacterial community structure. Indeed, *in vitro* bacterial competition assays revealed significant differences between the BFo1 strains in their ability to suppress growth of BFo2, and that these differences correlate with the ability of the latter to colonize WFT. Indeed, there is a 5-fold difference between the BFo1-H and BFo1-D strains in their ability to kill BFo2 bacteria. Conversely, there is 6-fold difference between the ability of BFo1-D to kill BFo1-H, compared to vice versa. This likely helps BFo1-D to displace BFo1-H in the Hereford WFT.

Mechanisms that can contribute to bacterial competition are multifaceted (Stubbendieck and Straight, 2016). For bean bugs, the natural symbiotic *Burkholderia* bacteria colonize midgut crypts, outcompeting other species in this specialized niche, likely through indirect mechansims (Itoh et al., 2019). Factors such as diet and the stage in the life-cycle of the host are other indirect mechanisms that can influence the structure of the associated microbiome, especially that of the insect gut (Douglas, 2015). The BFo1 genome possesses a Type VI secretion system (T6SS; Facey et al., 2015), and there are several examples that illustrate how these systems are used in bacterial competition (Hood et al., 2010; Murdoch et al., 2011), including how specific strains of *Vibrio fischeri* colonize light-organ crypts in squid species (Speare et al., 2018). However, as yet we have no experimental evidence to support a direct role for the BFo1 T6SS in the killing effects we have observed. Indeed, our attempts to analyse this system in the two BFo1 strains have been frustrated by a restriction barrier that, although not acting on small plasmids such as pdagGFP or pMK, prevents genetic manipulation with many other vectors. Both BFo1 strains can successfully kill *E. coli* to an even greater extent than they suppress BFo2. Analysis of the WFT genome has revealed no obvious homologs of the IMD gene, nor the FADD gene (Rotenberg et al., 2020). The former encodes a signal transducing molecule that acts in conjunction with FADD to activate transcription of antimicrobial peptide genes in response to Gram-negative pathogens, a well-conserved immune signaling pathway in insects (Palmer and Jiggins, 2015). The absence of this immune pathway in WFT and the killing effects of BFo1 suggest that the symbiont could play an important role in protecting its host from Gram-negative pathogenic bacteria acquired during feeding. Perhaps differences in community composition of these pathogenic bacteria in diverse geographical locations are a driver for BFo1 strain variance. The very high abundance of this gut symbiont suggests that the bacteria play a significant contribution to the fitness of their insect host.

This study indicates, for the first time, that strain differences between bacterial symbionts can play a large role in determining bacterial community structure in an insect’s GI tract. This is an important observation in terms of how paratransgenesis and, in particular, SMR can be applied to control pest insect populations such as WFT.

## Data Availability Statement

The original contributions presented in the study are included in the article/supplementary material, further inquiries can be directed to the corresponding author.

## Author Contributions

AA and MW: experimental analyses, data analysis, drafting manuscript, and figures. RS: data analysis and editing manuscript. MH: data analysis, drafting figures, and editing manuscript. PD: project management, experimental analyses, data analysis, and preparing manuscript. All authors contributed to the article and approved the submitted version.

## Funding

This project was funded by a grant from UKRI (BBSRC grant ref. BB/R006148/1) to PD. We also acknowledge the support of the Supercomputing Wales project, which is part-funded by the European Regional Development Fund (ERDF) *via* Welsh Government. Open access charges will be met by a UKRI block grant to Swansea University.

## Conflict of Interest

The authors declare that the research was conducted in the absence of any commercial or financial relationships that could be construed as a potential conflict of interest.

## Publisher’s Note

All claims expressed in this article are solely those of the authors and do not necessarily represent those of their affiliated organizations, or those of the publisher, the editors and the reviewers. Any product that may be evaluated in this article, or claim that may be made by its manufacturer, is not guaranteed or endorsed by the publisher.
